# A geospatial dataset providing first-order indicators of wildfire risks to water supply in Canada and Alaska

**DOI:** 10.1016/j.dib.2020.105171

**Published:** 2020-01-23

**Authors:** François-Nicolas Robinne

**Affiliations:** Canadian Partnership for Wildland Fire Science, Renewable Resources, 751 General Services Building, University of Alberta, Edmonton, T6G 2H1, Alberta, Canada

**Keywords:** Wildfires, Water supplies, Ecosystem services, Post-fire hydrology, Forest watershed, Watershed disturbance

## Abstract

First-order, high level indicators of wildfire risk to water resources are paramount to understand growing wildfire-related water security challenges in Canada and Alaska. Information pertaining to forest cover, fire activity, water availability, and location of populated places was collected from multiple institutional sources. Manual and semi-automated processes were used to clean disparate source data and create four harmonized geospatial layers whose content was summarized for each of the 1468 existing sub-sub watersheds covering Alaska and Canada. The final dataset provides a master layer based on sub-sub-watershed boundaries that contains relevant information to create spatial indicators of wildfire risk to water security. These can be used to identify potentially at-risk regions in high-latitude watersheds of North America. The dataset can be further used within a larger, general risk assessment framework considering other environmental stressors to water security, including climate change and population growth. The dataset described herein was used to make a figure in the manuscript “Wildfire impacts on hydrologic ecosystem services in North American high-latitude forests: A scoping review” by Robinne et al. [1].

**Table:** Specifications Table

Subject	Environmental sciences: Management, Monitoring, Policy and Law
Specific subject area	Wildfire risks to water security
Type of data	• Processed geospatial vectors and grids • Figure
How data were acquired	Source data were downloaded from institutional websites.
Data format	• Raw: ○ Populated places, wildfire perimeters, sub-sub watershed boundaries: vector (i.e., shapefile) ○ Snow water depth equivalent, Forest and shrubland cover: raster (i.e., tiff) • Cleaned/harmonized: ○ Populated places, wildfire perimeters, watershed boundaries: vector (i.e., ESRI geodatabase feature class) ○ Snow water depth equivalent, forest and shrubland cover: raster (i.e., ESRI geodatabase raster dataset) • Final (Master layer): vector (i.e., ESRI geodatabase feature class) • Figure: PNG image
Parameters for data collection	Data were selected based on the following parameters: • Availability in a GIS-ready format • Full coverage across Alaska and Canada • Available as single coverage (i.e., no province or county datasets to avoid tedious merging, to limit error risks, and to limit the multiplication of data sources) • Up-to-date information • Open-data access
Description of data collection	Data were searched for using the Google Search Engine, downloaded, and organized using ArcCatalog from ArcGIS Desktop 10.5 [10]
Data source location	Country: Canada, USA, and Italy
Data accessibility	• Raw data: ○ Sub-sub watershed boundaries (Canada): ▪ Repository name: Open Government ▪ Data identification number: a4b190fe-e090-4e6d-881e-b87956c07977 ▪ Direct URL to data: https://open.canada.ca/data/en/dataset/a4b190fe-e090-4e6d-881e-b87956c07977 ○ Sub-sub watershed boundaries (Alaska): ▪ Repository name: USGS The National Map ▪ Direct URL to data: http://prd-tnm.s3-website-us-west-2.amazonaws.com/?prefix=StagedProducts/Hydrography/WBD/National/GDB/o Fire perimeters (Canada): ▪ Repository name: Canadian Wildfire Information System Datamart ▪ Direct URL to data: http://cwfis.cfs.nrcan.gc.ca/datamart/download/lfdb?token=f321ac5752fa2a671d3fa42877a33b90 ○ Fire perimeters (Alaska): ▪ Repository name: MTBS Direct Download ▪ Direct URL to data: https://www.mtbs.gov/direct-download ○ Forest and shrubland cover: ▪ Repository name: FAO Geonetwork ▪ Direct URL to data: http://www.fao.org/geonetwork/srv/en/main.home?uuid=ba4526fd-cdbf-4028-a1bd-5a559c4bff38 ○ Snow water depth equivalent: ▪ Repository name: Goddard Earth Sciences Data and Information Services Center ▪ Data identification number: https://doi.org/10.5067/SXAVCZFAQLNO ▪ Direct URL to data: https://disc.gsfc.nasa.gov/datasets/GLDAS_NOAH025_M_V2.1/summary ○ Populated places (Canada): ▪ Repository name: Open Government ▪ Data identification number: e27c6eba-3c5d-4051-9db2-082dc6411c2c ▪ Direct URL to data: https://open.canada.ca/data/en/dataset/e27c6eba-3c5d-4051-9db2-082dc6411c2c ○ Populated places (Alaska): ▪ Repository name: AK State geo-spatial data clearinghouse ▪ Direct URL to data: http://asgdc.alaska.gov/#14 • Harmonized and final (Master) data: ○ Repository name: Mendeley Data ○ Data identification number: 10.17632/g8d734gyb8.1#folder-c0ceb2f3-1f4f-4321-8c36-cd1681777b82 ○ Direct URL to data: https://doi.org/10.17632/g8d734gyb8.1#folder-c0ceb2f3-1f4f-4321-8c36-cd1681777b82 • Figure: ○ Repository name: Mendeley Data ○ Data identification number: 10.17632/g8d734gyb8.1#folder-0e677696-a1a3-4fb4-8abe-d8263b661d81 ○ Direct URL to data: DOI: https://doi.org/10.17632/g8d734gyb8.1#folder-0e677696-a1a3-4fb4-8abe-d8263b661d81
Related research article	F. Robinne, D.W. Hallema, K.D. Bladon, J.M. Buttle, Wildfire impacts on hydrologic ecosystem services in North American high-latitude forests: A scoping review, J. Hydrol. 581 (2020) 124360. https://doi.org/10.1016/j.jhydrol.2019.124360, [1].

**Table:** 

**Value of the Data** • This dataset is useful as it provides a large-scale, continental overview of the first-order components of the wildfire risk to water security in Canada and Alaska; namely area burned, water availability, forest cover, and populated locations that might be impacted by watershed health impairments. • First-order risk components can beneficiate researchers, managers, and policy-makers involved in water resources management and the development of policies for watershed restoration and conservation. • The scale at which the dataset is provided can further help high-level understanding of climate change effect on the spatial pattern of wildfire risk to water security. It can also be used to target spatial subsets (e.g., Eastern Canada watersheds) where fine-scale, more complex analysis is necessary to understand and mitigate existing risk. • Information provided by this dataset is valuable as it offers a first large-scale perspective on a growing problem in North America, which can be considered in the development of future water resource management policies and watershed protection strategies.

## 1 Data

**Fig. 1:**
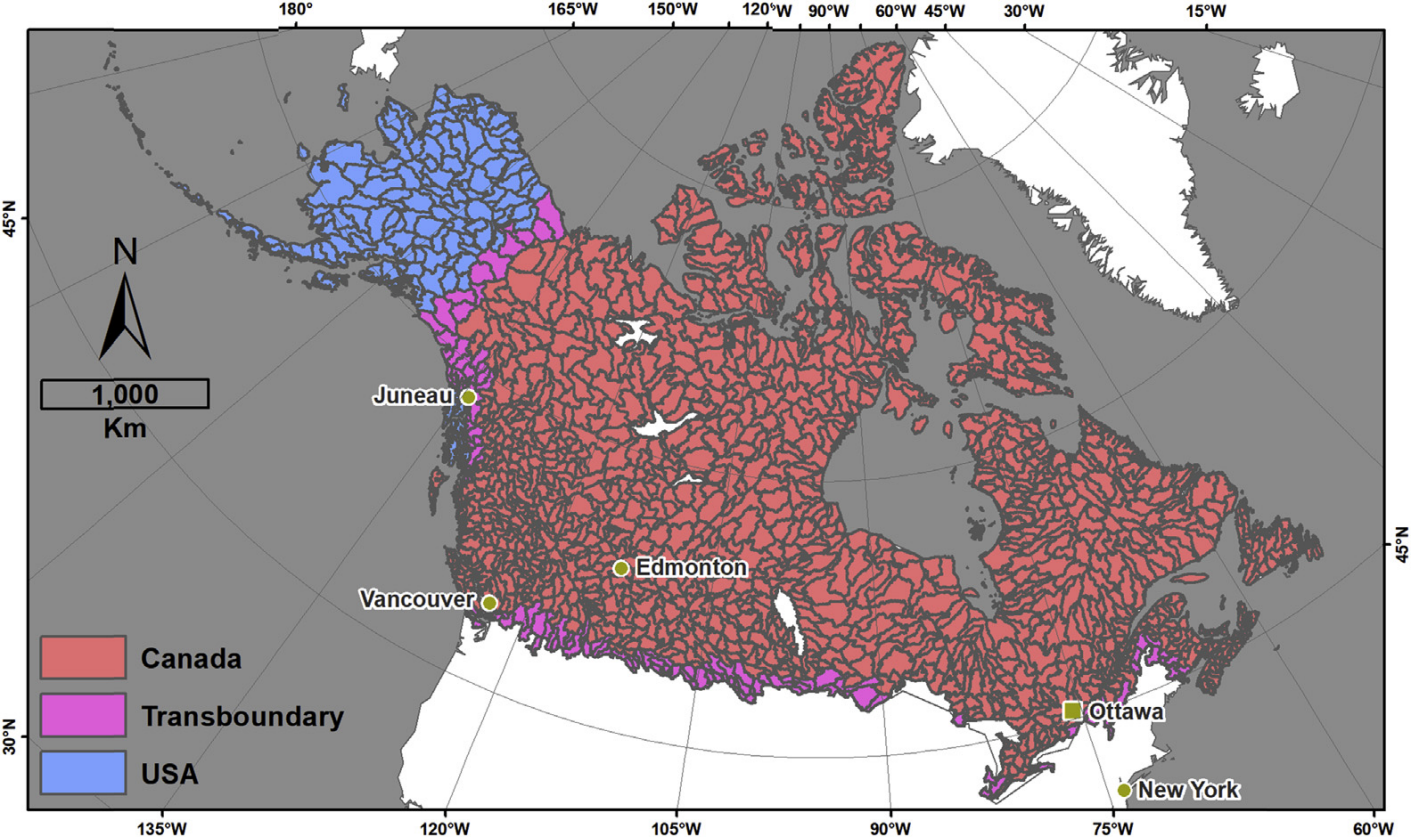
Sub-sub-drainages covering Alaska and Canada. The main feature class “HUC_SSD_CanAlaska_NAD83CSRS_Master” is projected in the NAD1983 CSRS Canada Atlas Lambert coordinate system. The layer contains 1468 polygons (single and multipart); 1235 are in Canada, 126 in USA (i.e. Alaska), and 107 are transboundary. Mean SSD size is ∼7527 km^2^, standard deviation is ∼5860 km^2^, and the range goes from ∼104 km^2^ to ∼54 500 km^2^.

**Fig. 2:**
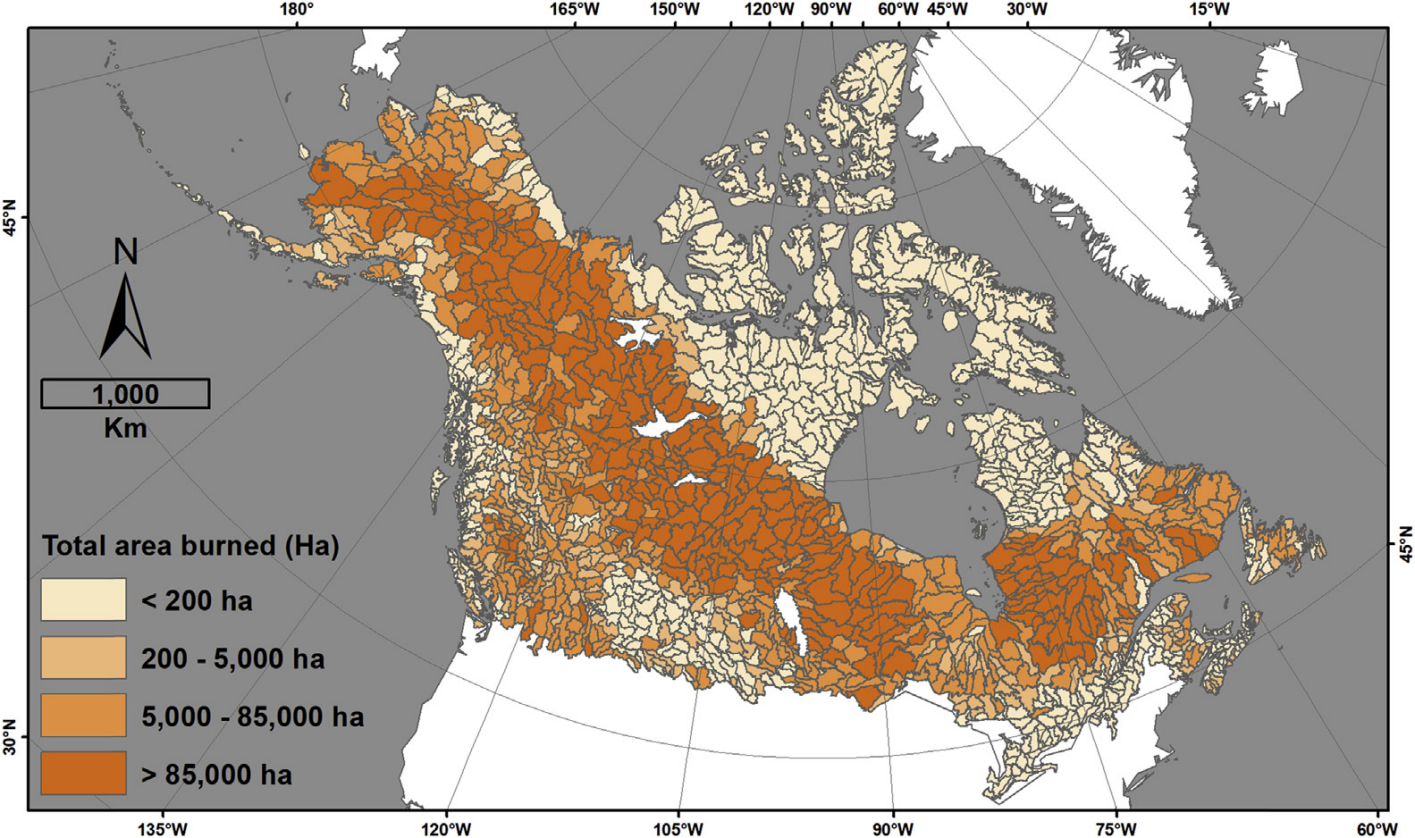
Area burned in hectares (ha) per sub-sub-drainages. The minimum value is 0; the maximum is 1 575 165.33; the average is 65408; the standard deviation is 162548; the sum is 96 020 371. Original information pertaining to fire perimeters is contained in the following feature class: CanAlaska_NAD83CSRS_FirePerimeters_Harmonized.

**Fig. 3:**
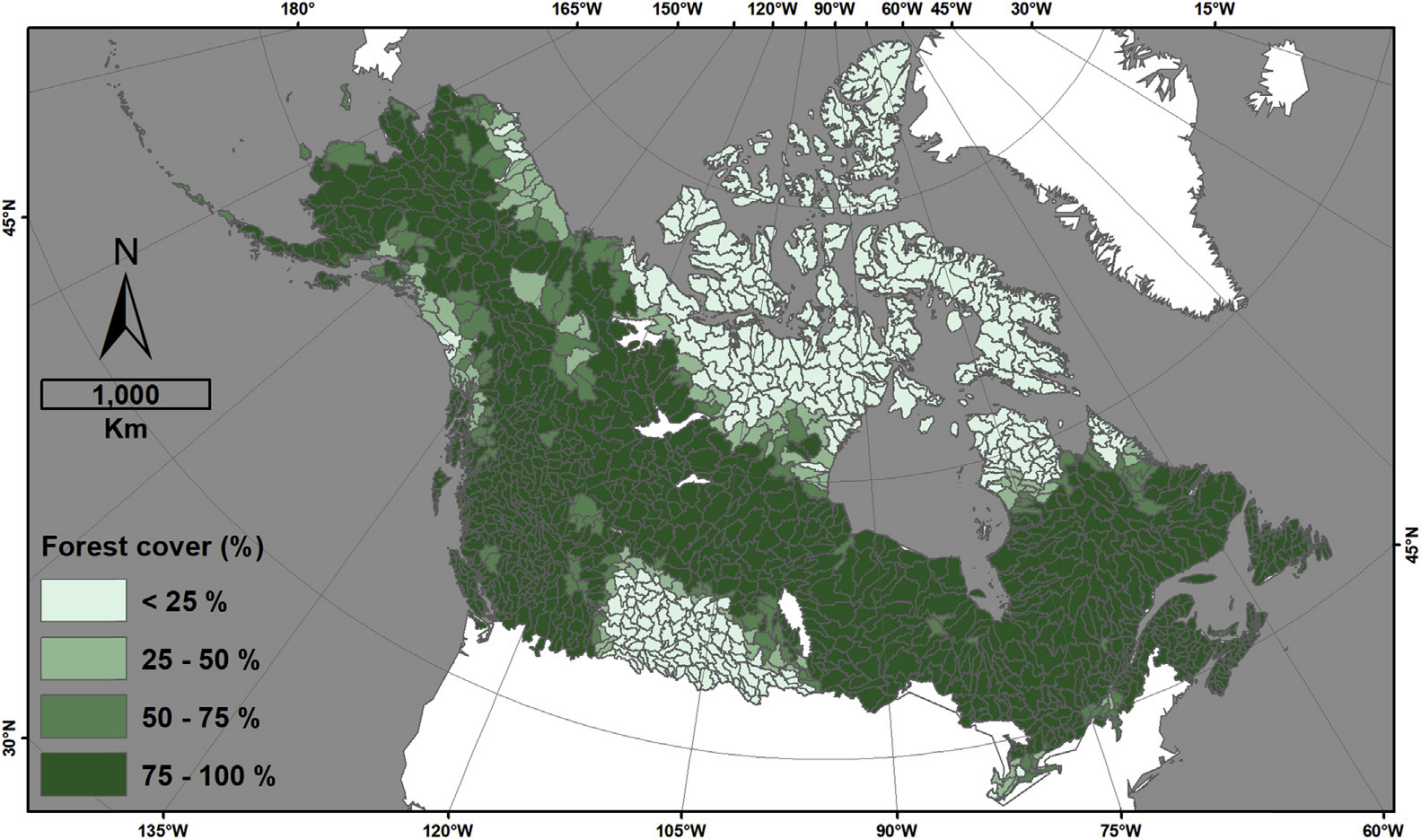
Percent (%) forest cover per sub-sub-drainage, an information also available as an area in square kilometers (km^2^). The minimum value is 0; the maximum is 100; the average is 68.83; the standard deviation is 36.8. Original information pertaining to forest land cover is contained in the following raster dataset: CanAlaska_NAD83CSRS_ForestCover_Harmonized.

**Fig. 4:**
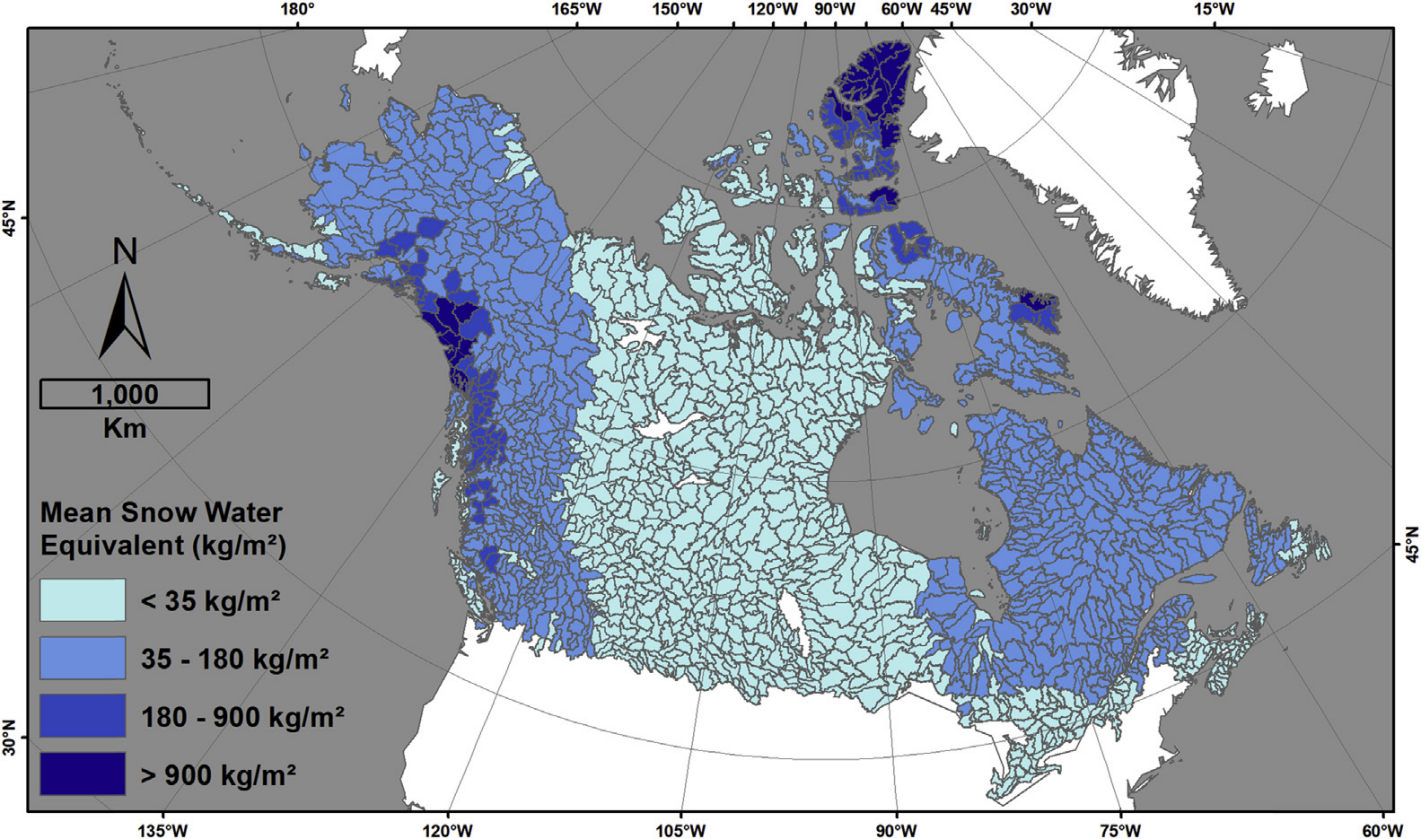
Mean snow water equivalent, in kilogram per square meter (kg/m^2^), per sub-sub-drainage. The minimum value is 0.55; the maximum is 4323; the average is 74.4; the standard deviation is 255; the sum is 108144.2. Original information pertaining to SWE cover is contained in the following raster dataset: CanAlaska_NAD83CSRS_SnowWaterEquivalent_Harmonized.

**Fig. 5:**
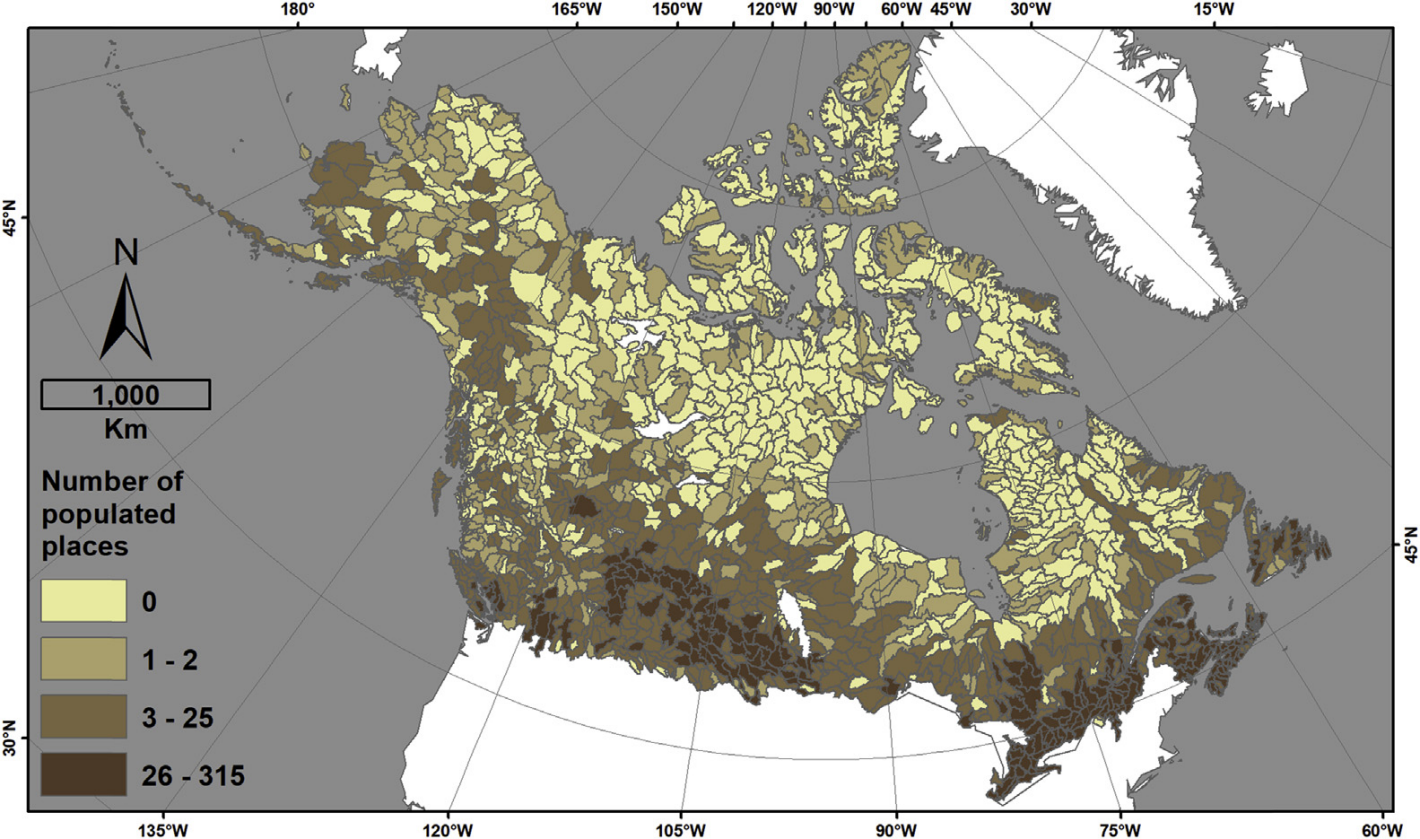
Number of populated places per sub-sub-drainage. The minimum value is 0; the maximum is 315; the average is ∼15, the standard deviation is 35, and the sum is 22543. The original information pertaining to the populated places is contained in the following feature class: CanAlaska_NAD83CSRS_PopulatedPlaces_Harmonized.

This data descriptor refers to Fig. 1 (hereafter, the figure) in Robinne et al. [1] and the associated dataset that was created to make the figure. The figure displays four different indicators for sub-sub drainages (SSD, equivalent Hydrologic Unit Code level 8 (HUC-8) in USA) of Canada and Alaska that are covered by a minimum of 30% forest; namely, percent forest cover, area burned normalized by forest cover, snow water depth equivalent, and the number of populated places. The associated dataset (CanAlaska_SubSubDrainages_RiskIndicators.gdb) provided in an ESRI geodatabase format is the product of spatial data combination from different public sources involving a significant amount of time in manual and semi-automated quality checks, updates, and harmonization. The dataset provides the main feature class in vector format (HUC_SSD_CanAlaska_NAD83CSRS_Master), whose attribute table contains complete hydrologic information pertaining to Canadian and Alaskan SSDs. In other words, it means that each SSD can be traced back to higher hydrologic levels and hydrologic regions (sub-drainage and main drainage), the country(ies), and the state/province(s)/territory(ies) they belong to (Fig. 1). The attribute table also contains information on total area burned in hectares (CanAlaska_NAD83CSRS_FirePerimeters_Harmonized) per SSD (Fig. 2), the percent forest cover (CanAlaska_NAD83CSRS_ForestCover_Harmonized) per SSD (Fig. 3), the average snow depth water equivalent (CanAlaska_NAD83CSRS_SnowWaterEquivalent_Harmonized) per SSD in meters (Fig. 4), and the number of populated places (CanAlaska_NAD83CSRS_PopulatedPlaces_Harmonized) per SSD Fig. 5). Main metadata for the dataset is contained at the root of the geodatabase.

## 2 Experimental design, materials, and methods

The creation of this dataset was fully based on the geoprocessing capabilities provided by ArcGIS 10.5 [10]. Considering the diversity of data sources and of their internal structure, it was not possible to fully automate data cleaning and aggregation; therefore, the preparation of the dataset described herein relied exclusively on manual editing and semi-automated updates using native tools available with an “advanced” ArcGIS license. Importantly, the level of methodological details provided below (i.e., type of processing tools and what they achieve) supposedly makes this dataset reproducible using any GIS freeware, such as QGIS.

### 2.1 Drainages

The creation of a seamless SSD layer started with the modification of the attribute table structure for both Alaskan and Canadian watershed layers [[2], [3]], thereby facilitating their merging. Relevant information pertaining to the state/province/territory, the country, the administrative code and the name of the different drainage levels, and the ocean the SSD drains into were kept and updated if necessary (e.g., adding missing information). Sub-drainage and drainage information were originally missing for HUC-8 watersheds in Alaska, and a spatial join was used to update the attribute table with the necessary details from higher hydrologic level layers (HUC-6 and HUC-4).

After merging both watershed layers, the attributes related to administrative location (i.e., country and province/state) were updated in a semi-automated way so the code of the administrative entity with the largest area covered by the SSD was added to the field “Administrative Entity – First”, in a decreasing fashion so “Administrative Entity – Fourth” would display the entity with the least area in the SSD. No SSD overlapped more than four administrative entities. In the case of an SSD overlapping international borders and existing in both source layers, the Canadian information was kept. For those drainages overlapping with the contiguous USA, administrative location attribute was left to 'US'. In total, 451 polygons needed their attributes to be manually updated with administrative information.

Further updates to the geometry were also necessary after merging. First, the SSD layer was clipped to the coastline, and the Great lakes were removed, as well as Great Slave Lake, Lake Winnipeg, Lake Athabasca, Great Bear Lake, and Lesser Slave Lake as those SSDs only displayed water. The layer was cleaned for slivers, gaps, micro-polygons, empty geometries, and self-intersections. The Integration Tool available in ArcGIS was used to make SSD boundaries snap to each other where minimal mismatches existed. Adjacent drainages with sizeable border mismatches were fixed manually. The Repair Geometry and Eliminate tools helped with empty geometries (n = 7) and micro-polygons (n = 3515).

**Fig. 6:**
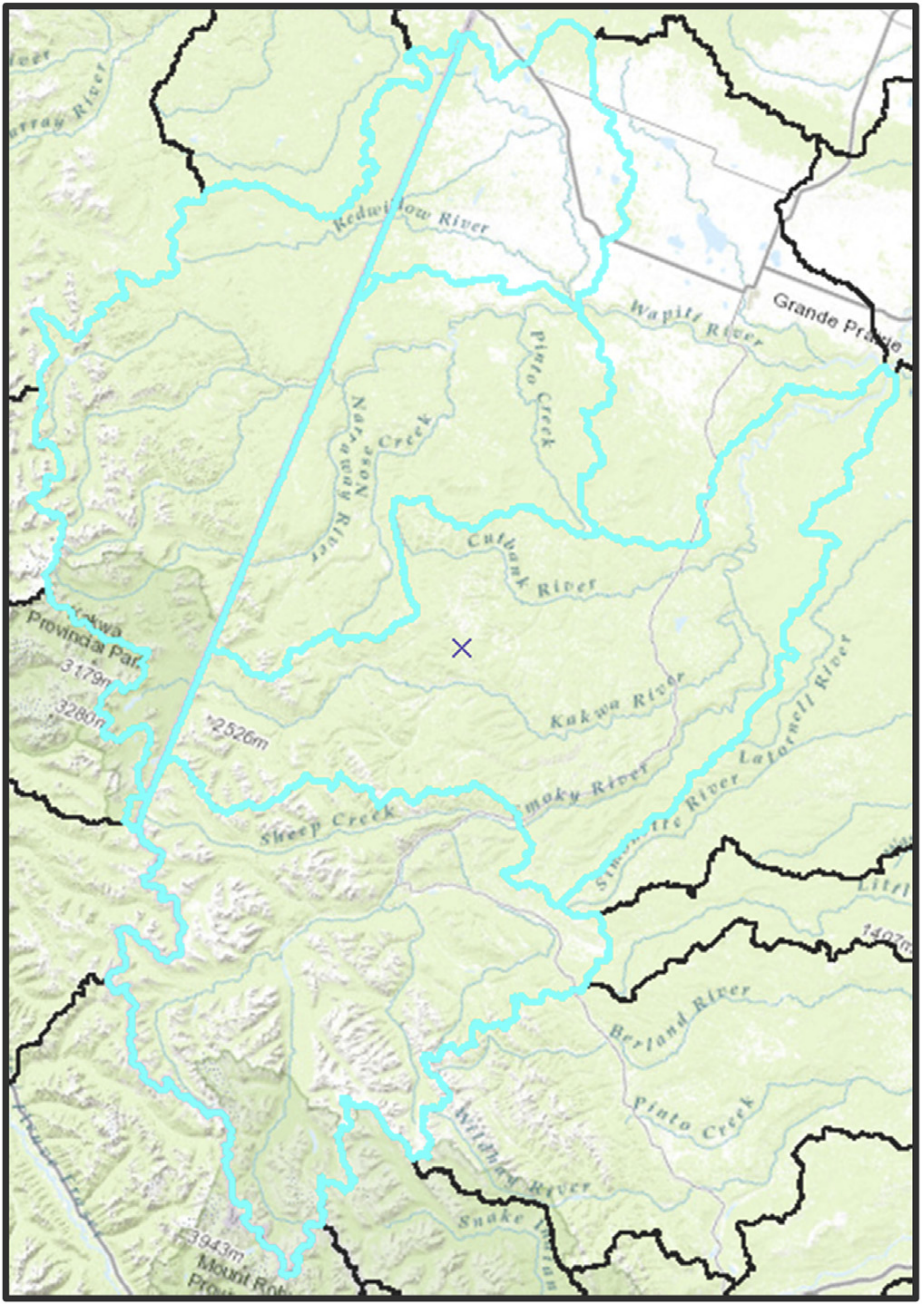
Example of boundary mismatch among several watersheds for which the province border between British Columbia and Alberta was not corrected to account to hydrologic continuity, thus displaying a straight line. In this case, the mismatch was left as is and the watersheds were flagged as erroneous in the database.

Then a topology layer was created to manually check and fix remaining overlap and gap errors. The layer had nearly 3800 overlap errors and 3400 gap errors to be cleaned. In some cases, discontinuities among adjacent SSD boundaries were impossible to fix without reprocessing drainage borders using GIS tools and thus were left as is (Fig. 6). Finally, a flag field was added; if coded 1, it would inform the user that there are doubts regarding the validity of the polygon geometry and/or the validity of its attributes. The final layer (HUC_SSD_CanAlaska_NAD83CSRS_Master) contained 1468 polygons.

### 2.2 Fire perimeters

Fire perimeters for Alaska were extracted from the whole MTBS dataset for USA, and all fire data available, from 1984 to 2016, was kept [4]. For Canada, only fire perimeters from 1980 to 2018 were kept, as fire data before this date are considered highly uncertain [[5], [6]]. It yielded 11 485 fire perimeters in Canada (out of 20 966) and 1386 perimeters for Alaska. Data structure was simplified then perimeter data was merged and spatial statistics were compiled per SSD to compute and store total area burned in hectares in the field “Total area burned (Ha)”.

### 2.3 Forest cover

Forest and woodland/shrubland land cover types from FAO land cover data [7] were merged into a single layer with a single coded value (i.e., 1). Using the Zonal Statistics tool in ArcGIS, the sum of forest cover was computed per SSD; as the pixel resolution of the source data equals 1 km, a sum automatically provides an area in square kilometers, information that was added to the attribute table in the field “Forest cover (km^2^)”. Then percent forest cover was computed using SSD area and added to the field “Forest cover (%)”. SSDs without forest cover were assigned an area value of 0, and SSDs with a percentage slightly over 100 (due to mismatches between 1km-sized pixels and SSD vector boundaries) were recoded to 100.

### 2.4 Populated places

Populated places data for Alaska was filtered so places with null population information were discarded, which left 280 locations out of 321 in the source data [8]. For Canada, populated places were extracted from a national dataset of points of interest, leading to a layer of 29 820 points out of 349 740 in the source data [9]. Data for Canada did not contain information regarding population count but a field provided enough detail to apply further filtering, so only places that were coded as permanently inhabited were kept and communities noted as “former” or “abandoned” were discarded, as well as non-community points such as post-offices, railway points, and resorts. The final layer for Canada contains a total of 23 811 points. Both data layers were then merged and point counts were computed per SSD using a spatial join and added to the field “Number of populated places”.

### 2.5 Snow water depth equivalent

Snow water depth equivalent (SWE) was computed for each grid cell from 2000 to 2019 using monthly averages in meters available over North America at 0.25°-pixel resolution [11]. The online application GIOVANNI was used for its capability to compile long-term averages from existing data hosted on NASA repositories [12]. Using the Zonal Statistics tool in ArcGIS, the mean SWE was computed per SSD and resulting information was added to the attribute table of the master layer to the field “Mean Snow Water Equivalent (m)”. Fifteen SSDs have <null> values due to their small size and the consequent mismatch with pixel centroids in the original SWE raster.

### 2.6 Limitations to usage

The dataset has three main limitations. Firstly, SSDs are not hydrologically connected, meaning that the layer cannot be used as is for hydrologic modeling purpose (e.g., downstream flow accumulation); however, both Canadian and Alaskan governments provide hydrographic networks whose information pertaining to upstream-downstream connectivity can be spatially connected to SSDs. Secondly, one can notice cases where area burned is greater than the forest in a given SSD (e.g., Central Souris - Moose mountain SSD), which is likely due to changes in land cover and/or the occurrence of prairie/rangeland fires that could have reached a significant size; therefore, working with those SSDs that have a forest cover greater than or equal to 30% is advised. Finally, the use of SSDs that were flagged for uncertainties in their name or boundaries should be avoided, or at least reported by the user.
